# Glucan Particles for Macrophage Targeted Delivery of Nanoparticles

**DOI:** 10.1155/2012/143524

**Published:** 2011-10-13

**Authors:** Ernesto R. Soto, Abaigeal C. Caras, Lindsey C. Kut, Melissa K. Castle, Gary R. Ostroff

**Affiliations:** Program in Molecular Medicine, University of Massachusetts Medical School, 373 Plantation Street, Worcester, MA 01605, USA

## Abstract

Glucan particles (GPs) are hollow, porous 2–4 **μ**m microspheres derived from the cell walls of Baker's yeast (*Saccharomyces cerevisiae*). The 1,3-**β**-glucan outer shell provides for receptor-mediated uptake by phagocytic cells expressing **β**-glucan receptors. GPs have been used for macrophage-targeted delivery of soluble payloads (DNA, siRNA, protein, and small molecules) encapsulated inside the hollow GPs via core polyplex and layer-by-layer (LbL) synthetic strategies. In this communication, we report the incorporation of nanoparticles as cores inside GPs (GP-NP) or electrostatically bound to the surface of chemically derivatized GPs (NP-GP). GP nanoparticle formulations benefit from the drug encapsulation properties of NPs and the macrophage-targeting properties of GPs. GP nanoparticle formulations were synthesized using fluorescent anionic polystyrene nanoparticles allowing visualization and quantitation of NP binding and encapsulation. Mesoporous silica nanoparticles (MSNs) containing the chemotherapeutic doxorubicin (Dox) were bound to cationic GPs. Dox-MSN-GPs efficiently delivered Dox into GP phagocytic cells resulting in enhanced Dox-mediated growth arrest.

## 1. Introduction

The development of effective drug delivery systems presents multiple challenges, such as issues of drug solubility, targeting, *in vivo* stability and clearance, and toxicity. Nanotechnology-based drug delivery systems are a promising approach to fulfill the need for new delivery systems offering several advantages, such as high drug binding capacity due to their large surface area, improved solubility and bioavailability of hydrophobic drugs, extended drug half-life, improved therapeutic index, reduced immunogenicity, and the possibility for controlled release [1–3]. Nanoparticles can also be synthesized with control over average size, size distribution, and particle shape, all key factors related to cellular uptake mechanisms and improved penetration across biological barriers. Additionally, some nanoparticles offer the possibility for combined use as therapeutic and diagnostic/imaging tools. A new term, theranostics, has been recently proposed to describe these types of nanoparticles [4]. The successful development of nanoparticle-based delivery systems is exemplified by the use of nanomaterials for anticancer drug formulations [5, 6]. 

A primary challenge to realizing the full promise of nanoparticle-based drug delivery is the lack of optimal strategies to achieve selective and efficient cellular targeting. The mechanism of NP uptake is dependent on particle size and shape [7–9], and several competing uptake mechanisms result in undesired processes including off-target accumulation in other organs tissues and cells, rapid clearance from *in vivo* circulation (especially NPs less than 5 nm) [10, 11], opsonization and macrophage clearance [7, 12], and complement activation by proteins that results in hypersensitivity reactions [13]. NPs can be somewhat targeted by attaching ligands with specificity to receptors that are overexpressed in certain cells (i.e., folate and transferrin receptors in cancer cells [14–18]), or targeting cell populations with high selectivity by grafting specific targeting moieties to cell surface receptors known to be expressed only on target cells (i.e., antibodies to target prostate-specific membrane antigen (PSMA) [19] or galactose to target asialoglycoprotein receptors on hepatocyte cells [20]). Some interfering processes can be reduced by coating of the nanoparticles with a hydrophilic polymer (i.e., PEG polymer brush or stealth nanoparticles). PEG is a nonimmunogenic, nontoxic, and protein-binding resistant polymer. PEG coating of nanoparticles prevents opsonization by shielding surface charges, reducing macrophage clearance, increasing steric repulsion of blood components, and increasing hydrophilicity and *in vivo* circulation of NPs [12, 21].

Glucan particles (GPs) are porous, hollow microspheres that are prepared from *Saccharomyces cerevisiae* (Baker's yeast). The glucan microspheres have an average diameter of 2–4 microns and are composed of 1,3-D-glucan and trace amounts of chitin. The 1,3-D-glucan polysaccharide on the GP surface serves as a ligand for receptor-mediated cell uptake by phagocytic cells bearing *β*-glucan receptors (dectin-1 (D1) receptor and complement receptor 3 (CR3)) [22], such as macrophages and dendritic cells in the immune system. GP uptake has been demonstrated to be dectin-1 dependent *in vitro* [23]. The ability to selectively target phagocytic cells makes the glucan particle an attractive drug delivery vehicle for this cell population. The hollow and porous material properties of GPs allow for the encapsulation, transport, delivery, and release of electrostatically bound payloads. Previously we have reported the use of GPs for macrophage-targeted delivery of soluble payload macromolecules (i.e., proteins [23], DNA [24], and siRNA [25, 26]), and small drug molecules, such as the antibiotic rifampicin [27]. However, the use of GPs for small drug molecule delivery is limited since the majority of small drug molecules are neutral, monovalent in charge, or insoluble in water, and such payloads are not easily trapped within glucan particles using the polyplex core or LbL encapsulation methods developed for nucleic acids and proteins. 

**Figure 1:**
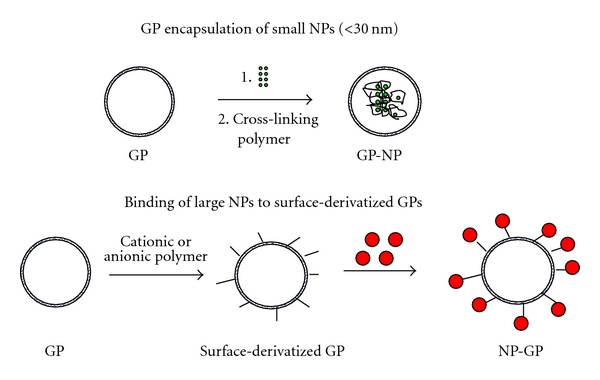
Schematic representation of glucan particle/nanoparticle synthesis strategies.

We report here a new targeted-nanoparticle delivery application for glucan particles incorporating insoluble preformed nanoparticles (NPs) of less than 30 nm in diameter as cores inside glucan particles (GP-NP), or nanoparticles electrostatically bound to the surface of derivatized glucan particles (NP-GP) (Figure 1). The advantages of GP nanoparticle encapsulation include: (1) glucan receptor targeted delivery (2) the encapsulation of payload complexes that cannot be prepared *in situ* as the synthetic conditions are not compatible with glucan particles, (3) the loading of nanoparticles that can enhance the ability to load small drug molecules (neutral, hydrophobic drugs) into GPs, and (4) the incorporation of nanoparticles with an intrinsic property, such as magnetic nanoparticles, thus increasing the versatility of the particles, as the same formulation could be used for drug delivery and the magnetic properties employed for cell purification or imaging applications [28].

The development of GP nanoparticle-loaded formulations used two types of model nanoparticles: (1) fluorescent polystyrene nanoparticles of narrow size distribution to allow for the visualization and characterization by fluorescent techniques and (2) mesoporous silica nanoparticles (MSNs) for the encapsulation of the chemotherapeutic drug, doxorubicin to assess biological activity. MSNs are highly porous nanoparticles prepared from tetraethyl orthosilicate polymerized on a template such as a surfactant micelle [29, 30]. Since the discovery of MSNs in 1992, much work has been done to evaluate these materials for absorption, catalysis, chemical devices, and more recently as drug delivery agents (i.e., delivery of the cancer drugs camptothecin, paclitaxel, doxorubicin [31–33]). MSNs were chosen as a model nanoparticle because of their ease of synthesis, binding capacity for small drug molecules, and the possibility of extending the capabilities of GP-targeted drug delivery to hydrophobic drugs. We chose to study the delivery of doxorubicin (Dox) as a first step in the development of GP macrophage-targeted delivery of chemotherapeutics. Macrophages are nondividing differentiated cells and are resistant to the cytotoxic DNA replication inhibitor, doxorubicin. Macrophages are known to migrate into solid tumors, and we hypothesize that they will act as Trojan horses carrying lethal doses of Dox-GPs into tumors for targeted cancer drug delivery to rapidly dividing tumor cells.

## 2. Materials and Methods

### 2.1. Materials

Carboxylate-modified red fluorescent nanoparticles and Alamar blue were purchased from Invitrogen (Carlsbad, CA); glucan particles (GP) were prepared from Baker's yeast (Fleishmans Baker's yeast, AB Mauri Food Inc, Chesterfield MO, USA) according to a previously published procedure [24]. Polyethylenimines (PEIs, molecular weight of 1.2, 10, and 100 kDa) were purchased from Polysciences (Warrington, PA, USA). All other PEIs, chemicals for the synthesis of MSNs, and solvents were purchased from Sigma Aldrich (Allentown, PA, USA) and used as received. Materials for cell tissue culture experiments were purchased from Gibco Scientific (Grand Island, NY, USA) or Fisher Scientific (Fairlawn, NJ, USA).

### 2.2. Preparation of 20 nm Nanoparticle Cores Inside GPs

Fluorescent 20 nm nanoparticles were used at a concentration of 4.5 × 10^15^ particles/mL. Dry glucan particles were mixed with 5 *μ*L nanoparticle suspension/mg GP to obtain a uniform paste, incubated at room temperature for 1 h, and the GP-NP-loaded formulation was lyophilized. This hydration-loading-lyophilization procedure was repeated using water (5 *μ*L/mg GP) to hydraulically push nanoparticles into GPs by capillary action. The dry GP-NPs were hydrated and washed after the second lyophilization to remove free nanoparticles, sterilized in 70% ethanol at −20°C, aseptically washed three times with 0.9% saline, resuspended in 0.9% saline, counted with a hematocytometer, and GP concentration adjusted to a concentration of 1 × 10^8^ particles/mL. Samples were evaluated by fluorescence microscopy, flow cytometry, zeta potential, and for GP-mediated uptake into phagocytic cells.

### 2.3. Synthesis of Cationic GPs

GPs (5 mg) were resuspended in 10 mLs of water by homogenization. Potassium periodate (0.4 mL of a 1 mg/mL solution) was added and the mixture stirred in the dark at room temperature for at least six hours. Oxidized GP samples were washed three times with water, and used immediately for reductive amination synthesis. Cationic polymers (PEIs) and water were added to the oxidized GP samples (1 *μ*mol PEI/mg GP), and the particles were resuspended and mixed at room temperature overnight. The aminated samples were reduced with sodium borohydride (0.1 g) and incubated at room temperature for 48 hours. The reduced samples were washed with water. Tris buffer (5 mLs, pH 7.5, 0.05 M) was added and the sample incubated for 30 minutes. The samples were washed with water, resuspended in 70% ethanol, and stored overnight at −20°C for sterilization, then aseptically washed three times with 0.9% saline, resuspended in 0.9% saline, particles counted with a hematocytometer, and the particle suspensions diluted to a concentration of 1 × 10^8^ particles/mL and stored at −20°C. 

Periodate oxidized glucan particles (*n* = 5 samples) were evaluated for aldehyde content using a hydroxylamine hydrochloride assay [34]. Oxidized GP samples (5 mg) were incubated in 1 mL of DMSO at 50°C for 2 h to dissolve the particles. The samples were centrifuged to remove insoluble material (chitin). Hydroxylamine hydrochloride solution (0.5 mL, 0.5 N) containing 0.05% w/v methyl orange added to the samples, and the mixture was incubated at room temperature for 4 hours. The samples were titrated with a standardized 0.01 M sodium hydroxide solution until a red-to-yellow endpoint was achieved. 

The level of PEI coupling in the PEI-GP samples was measured with a ninhydrin assay. PEI-GP samples (1 mg) were resuspended in 100 *μ*L of water and mixed with 100 *μ*L of 2% w/v ninhydrin in DMSO. The samples were heated at 100°C for 20 min, cooled to room temperature, and 800 *μ*L of ethanol was added. PEI samples of different concentrations were also treated with ninhydrin to prepare calibration curves and determine the linear response range of each of the PEIs used in the chemical modification of GPs. Absorbance was measured at 570 nm for the calibration curve controls, PEI-GP samples, and blank GP controls. A total of three samples of each PEI-GP were analyzed with the ninhydrin assay.

### 2.4. Binding of Nanoparticles to Surface Derivatized GPs

GP or PEI-GP samples (10 *μ*L 1 × 10^8^ part/mL) and rhodamine labeled carboxylated nanoparticles of different diameter (20, 100 and 200 nm) were mixed at NP/GP ratios of 1/1, 10/1, and 100/1 in a final volume of 100 *μ*L in 0.9% saline. The samples were incubated in the dark for 1 hour and the unbound nanoparticles separated from the GPs containing bound nanoparticles by centrifugation (10000 rpm for 2 min). The samples were then washed with 0.9% saline (100 *μ*L) to remove unbound nanoparticles from the pellet, washed pellet samples resuspended in 0.9% saline (100 *μ*L) and fluorescence of the carboxylate polystyrene nanoparticles (excitation = 580 nm, emission = 605 nm) measured in all fractions to quantify bound and unbound nanoparticles. The average of at least five measurements was collected for each experimental condition. NP-GP samples were also evaluated by zeta potential measurements, flow cytometry, and fluorescence microscopy. The NP-GP samples were tested for nanoparticle-binding stability to GPs by incubation in phosphate buffer saline (PBS, pH 7) containing 10% fetal bovine serum (FBS), or sodium acetate buffer (0.1 M, pH 5) over 48 hours. Samples were processed by centrifugation to remove free nanoparticles, washed and supernatant fractions analyzed for released cPS-NPs, and pelleted fractions evaluated by fluorescence microscopy to assess binding of cPS-NPs to GPs.

### 2.5. Mesoporous Silica Nanoparticles (MSNs)

MSN samples containing phosphate and amine functional groups were prepared by the cocondensation method reported by Lu et al. [32, 33]. A solution containing cetyl trimethylammonium bromide (CTAB, 0.5 g) in water (24 mLs), and NaOH (2 M, 0.2 mL) was heated to 80°C and stirred vigorously until the solutes were dissolved. A solution containing the MSN reagents (tetraethylorthosilicate, TEOS (2.5 mLs) and amino-propyltriethoxysilane, APTS (12 *μ*Ls)) were then added and the mixture stirred at 80°C for 15 minutes. 3-Trihydroxysilylpropyl methylphosphonate (0.63 mL) was added and the solution was incubated for 2 hours at 80°C with stirring. The solution was cooled at room temperature and then centrifuged (3,000 g for 20 minutes), washed with 50 mLs of methanol, and dried at room temperature. The CTAB was extracted from the MSN by refluxing the particles (850 mg) in an acidic methanol mixture (90 mLs of methanol and 5 mLs of 12.1 M HCl) for 24 hours. The particles were then washed three times with 50 mLs of methanol and left to dry overnight. MSN samples were characterized by dynamic light scattering (DLS) particle size measurements, and zeta potential.

### 2.6. Doxorubicin Binding to MSN

MSN suspensions and doxorubicin (0–2 *μ*mol Dox/mg MSN) were incubated in 1 mL of DMSO overnight at room temperature. The samples were then centrifuged and the supernatant removed. The Dox-MSN pellets were lyophilized and washed three times with 1 mL of water to remove Dox bound on the outside of MSN. A total of five Dox-MSN samples were prepared for each Dox loading concentration. Dox-MSN samples were resuspended in sterile water at a concentration of 1 mg/mL and stored at −20°C. The amount of Dox bound to MSN was quantified by incubating 0.2 mg samples of Dox-MSN in methanol (1 mL) overnight at room temperature to completely extract Dox. Doxorubicin was quantified by fluorescence spectroscopy (excitation = 480 nm, emission = 550 nm).

### 2.7. Synthesis of Dox-MSN-PEI-GP

25 k PEI-GPs, unmodified GPs (10 *μ*L, 1 × 10^8^ particles/mL), and Dox-MSN nanoparticles (10 *μ*L, 5 × 10^−5^ to 5 × 10^−2^ mg Dox-MSN/mL) were mixed at a final volume of 100 *μ*L in 0.9% saline. The concentration range of Dox-MSN allowed studying binding to PEI-GP at Dox-MSN/PEI-GP ratios from 0.0005 to 5 pg Dox-MSN/GP. The samples were incubated in the dark for 1 hour at room temperature and the unbound Dox-MSN separated from the PEI-GP or GPs-containing bound nanoparticles by centrifugation (10000 rpm for 2 min). Additional control samples containing only Dox-MSN were processed in the purification procedure. The samples were then washed with 0.9% saline (100 *μ*L) to remove unbound nanoparticles from the pellet, washed pellet samples resuspended in 0.9% saline (100 *μ*L), and fluorescence of doxorubicin measured in all fractions to quantify amount of bound and unbound Dox-MSN. The average of at least three measurements was collected for each experimental condition. Dox-MSN-PEI-GP and Dox-MSN-GP samples were also evaluated by zeta potential measurements, and fluorescence microscopy. The samples were evaluated for stability by incubation in phosphate buffer saline (PBS, pH 7) containing 10% fetal bovine serum (FBS), or sodium acetate buffer (0.1 M, pH 5) over 48 hours. At various time points, samples were processed by centrifugation to remove free nanoparticles, washed and supernatant fractions analyzed by fluorescence spectroscopy to quantify released Dox, and pellet fractions were analyzed by fluorescence microscopy to confirm stability of Dox-MSN-PEI-GP samples.

### 2.8. Dynamic Light Scattering (DLS) and Zeta Potential Measurements

Size and zeta potential of nanoparticle samples and zeta potential of GP/NP samples were determined with a Malvern Zetasizer Nano-ZS (Malvern Instruments, Worcestershire, UK). Solvents and buffers were filtered through 0.22 *μ*m filters before sample preparation. A suspension of particles (1 mg/mL for nanoparticle samples, 2 × 10^6^ particles/mL for GP samples) was diluted in 1 mL of 20 mM Hepes buffer, vortexed, and transferred to a 1 mL clear zeta potential cuvette (DTS1061, Malvern). Zeta potential was collected at 25°C from −150 to +150 mV. The results are the average three samples. For each sample a total of 30 measurements were collected and analyzed with the Dispersion Technology software 4.20 (Malvern) producing diagrams of zeta potential distribution versus total counts. DLS measurements were obtained from samples in the same zeta potential cells at 25°C. The average of 20 measurements/sample was collected in the size range from 1 nm to 10000 nm. The data were analyzed with the Dispersion Technology software producing histograms for particle size versus % intensity.

### 2.9. Flow Cytometry (FACS)

FACS measurements were obtained using a Becton Dickinson FACSCalibur instrument (BD, Franklin Lakes, NJ, USA). Samples were prepared for FACS analysis by binding of 2 × 10^7^ nanoparticles to 2 × 10^6^ GP particles. The bound NP-GP samples were washed from unbound nanoparticles and resuspended at 2 × 10^6^ GP/mL in PBS. Unmodified GPs were used as negative control and rhodamine-labeled GPs as the positive control. The particles were analyzed with an FL4 laser at 605 nm by collecting an average of 15000 measurements. Gating and analysis was performed using FlowJo 6.4.2 software.

### 2.10. Dox-MSN/GP Cell Delivery

Dox-MSN samples were prepared as described in Section 2.6. Dox-MSN samples were bound to 25 k PEI-GP or unmodified GP particles as described in Section 2.7. The samples were prepared by binding 0–5 × 10^−4^ mg Dox-MSN/1 × 10^6^  PEI-GP or GPs, equivalent to 0–5 pg Dox-MSN/glucan particle. The amount of PEI-GP or GP particles was chosen to test for cell uptake at a 10 : 1 GP : cell ratio to maximize phagocytic cell uptake. Based on the binding of Dox to Dox-MSN, the Dox-MSN-PEI-GP samples contained 0–0.15 nmol Dox. Dox-MSN-free nanoparticles and soluble Dox (free Dox) were also evaluated in the same concentration range. These samples were evaluated for cellular uptake and Dox delivery using the NIH3T3-D1 cell line. This cell line was derived from the NIH3T3 fibroblast cell line by the integration of the dectin-1 gene to produce cells expressing the *β*-1,3-D-glucan receptor dectin-1 allowing for efficient GP phagocytosis [35, 36]. Samples were resuspended in complete DMEM medium (250 *μ*L) and added to 24 well plates containing 1 × 10^5^ cells in 0.5 mL complete DMEM medium. After incubation for 3 hours at 37°C under 5% CO_2_, the cells were fixed with 1% formalin and observed microscopically for fluorescent Dox-MSN/PEI-GP phagocytosis. To determine the effects of Dox-MSN/PEI-GP, Dox-MSN, or free Dox on cell growth and viability, these samples were incubated for 3 hours with cells as described above, and the cell monolayers were washed in complete DMEM and incubated for an additional 48 hours. Alamar blue (50 *μ*L) was added, the cells incubated at 37°C for 2 hrs, and fluorescence was measured, excitation wavelength = 530 nm, emission wavelength = 590 nm. Fluorescent response is dependent on the reduction of the Alamar blue indicator by metabolically active cells and is an indicator of cell number and viability. Growth arrest was calculated from the fluorescence response of the sample wells relative to the response of control wells containing cells incubated in the absence of doxorubicin. The results are the average of four samples prepared for each Dox-MSN formulation evaluated for NIH3T3-D1 growth arrest.

## 3. Results and Discussion

Glucan particles have been used for macrophage-targeted delivery of a wide range of payload macromolecules [23–27]. Soluble payloads can be efficiently encapsulated inside GPs by both polyplex and layer-by-Layer (LbL) synthetic approaches. There is a growing interest to extend the use of the GP delivery technology for small drug molecules (i.e., chemotherapeutics and antibiotics). However, GPs have limitations in the encapsulation of small molecules as most of these molecules are neutral in charge and cannot be trapped by the noncovalent techniques used to assemble macromolecule polyplexes inside GPs. Also, hydrophobic drugs present a challenge for loading inside GPs. The combination of established nanoparticle encapsulation technologies and glucan particles offers an attractive opportunity to extend the use of GPs for macrophage-targeted delivery of small drug molecules. Here we present the results of model systems using polystyrene nanoparticles and mesoporous silica nanoparticles to demonstrate the GP-mediated small drug molecule delivery as GP-nanoparticle-drug formulations. 

### 3.1. Use of Glucan Particles for Encapsulation or Surface Binding of Polystyrene (PS) Nanoparticles

**Figure 2:**
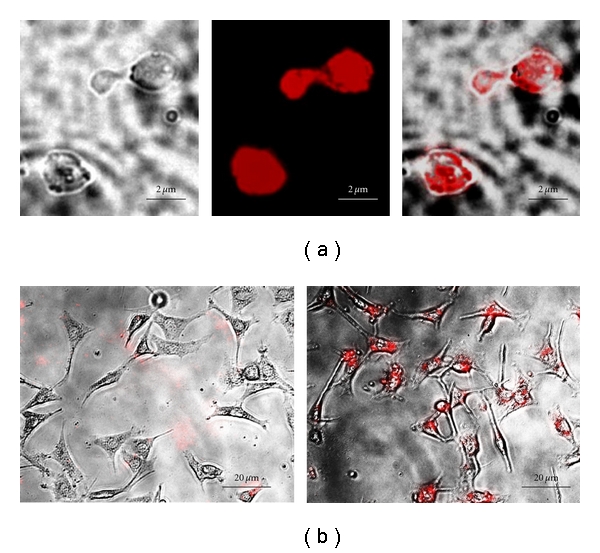
(a) Microscopic images of GPs containing 20 nm anionic fluorescent carboxylate polystyrene nanoparticles. (b) Fluorescent photomicrographs showing uptake of GP-cPS-NPs by control NIH3T3 fibroblast cells (left) and GP phagocytosing proficient NIH3T3-D1 cells (right).

Rhodamine-labeled carboxylate polystyrene nanoparticles (cPS-NPs) were used as a model system because of their uniform narrow size distribution, high fluorescent signal, and the ability to cross-link or bind carboxylate nanoparticles to the surface of cation-modified GPs through electrostatic interactions. cPS-NPs (20 nm in diameter) were used to prepare nanoparticle cores encapsulated within the hollow cavity of GPs. The freeze-thaw cycles during the nanoparticle-loading process caused nanoparticle aggregation trapping the cPS-NPs inside GPs. Additionally, the inclusion of a cationic polymer, like polyethylenimine (PEI) or chitosan, electrostatically crosslinked the aggregated nanoparticles inside GPs and slowed down their release.  Figure 2 shows microscopic images of GP nanoparticle cores and receptor-targeted uptake by cells bearing glucan receptors. cPS-NPs were loaded at a concentration of 2.25 × 10^13^ nanoparticles/mg GP, and measurement of unbound cPS-NPs collected from washing the GP-cPS NP cores demonstrated that nanoparticle encapsulation efficiency was greater than 80%. The high cPS-NP encapsulation capacity in the hollow cavity in GPs results in the loading of >30,000, 20 nm cPS-NPs per glucan particle. However, there is a limit to the size of nanoparticles that can be encapsulated inside GPs because the average pore size in the shell of GPs is less than 40 nm. To overcome this size limitation, an alternative approach was devised to bind nanoparticles to the outer GP shell. 

Cationic GPs were synthesized by functionalization of the GP surface with branched PEIs varying in molecular weight. The cationic PEI-GP library was prepared by reductive amination of oxidized glucan particles with PEI following similar procedures reported for other polysaccharides [37]. The 1,3-glycosidic bonds are stable to oxidation; thus oxidation of glucan particles takes place only at the reducing terminal glucose monomers (<2%) in the *β*-glucan structure of the particles. This limits the grafting of PEIs to ~0.12 *μ*mol PEI/mg GP. The yield of periodate oxidation of the terminal glucose in the particles (60 ± 10%) was determined using a hydroxylamine hydrochloride assay. This oxidation step limits the reaction of cationic PEIs by reductive animation to less than 0.07 *μ*mol PEI/mg GP. Binding of the cationic polymers to the glucan particles was confirmed by a ninhydrin test. The results of PEI grafting shown in Table 1 confirmed that the levels of PEI covalently linked to GPs ranged from 0.01 to 0.03 *μ*mol PEI/mg GP (15–40% yield based on a maximum PEI grafting of 0.07 *μ*mol PEI/mg GP). The molecular weight of PEI does not seem to have an effect on grafting; therefore, it is likely that accessibility of the oxidized glucose units on the particle surface is the controlling parameter in the reaction.

Zeta potential results (Table 1) confirmed the synthesis of cationic GPs. Zeta potential has been previously used to follow reaction sequences on nano- and microparticles [38]. Unmodified GPs are neutral and a significant shift to a positive potential demonstrated PEI linkage to GPs. One limitation of zeta potential measurements is the effect of particle aggregation on zeta potential to establish a quantitative relation between the number of surface groups and the zeta potential values. The zeta potential results of PEI-GP samples indicate that enough PEI has been grafted on the GP surface to shift the zeta potential of neutral GPs to ~20 mV (low molecular weight PEI) or ~30 mV (high molecular weight PEI).

Fluorescent anionic carboxylate polystyrene nanoparticles (cPS-NPs) of three diameters (20, 100, and 200 nm) were used to measure the binding capacity of PEI-GPs and control GPs. Larger nanoparticles (0.5, 1, and 2 um) were also evaluated, but quantitative analysis was difficult due to spontaneous aggregation of cPS-NPs with the GPs. cPS-NP-loaded GP samples prepared with nanoparticles of 200 nm or less in diameter can be separated from GPs by centrifugation. The amount of cPS-NPs bound to the GPs was measured from the fluorescence of unbound cPS-NPs collected in the supernatant and the bound nanoparticles in the NP-GP pellet fractions. The binding efficiency is defined as the ratio of fluorescence emission measurements of cPS-NPs in the pellet fraction divided by the input cPS-NPs.

(1) $$\frac{\text{Measured cPS-NP fluorescence in pellet}}{\text{input}}=\text{binding efficiency}$$

**Figure 3:**
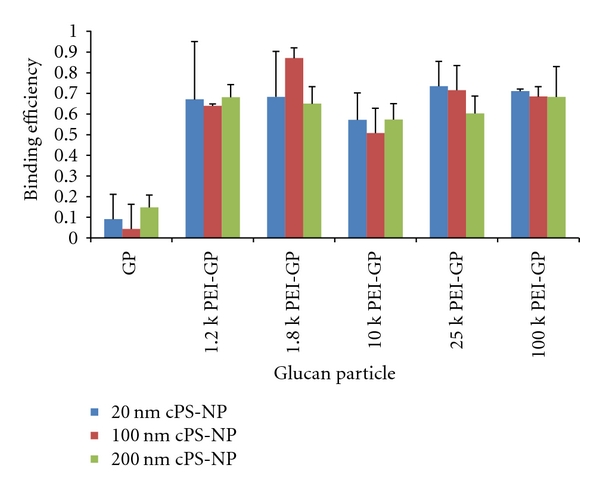
Binding efficiency of GPs derivatized with different molecular weights of PEI for anionic fluorescent polystyrene nanoparticles of 20, 100, and 200 nm in diameter (experimental results were obtained at a cPS-NP/GP ratio of 100 : 1, the values correspond to average of at least five samples).

Figure 3 shows that the PEI-GPs readily bind cPS-NPs. The binding efficiency of PEI-GPs for fluorescent anionic polystyrene nanoparticles was carried out at a ratio of 100 : 1 cPS-NPs : glucan particle and ranged from 50 to 90%. The unmodified GP control had minimal cPS-NP binding. The content of amines/GP increases with PEI molecular weight; however, there is no correlation between amine content (surface charge) and binding efficiency indicating that at the cPS-NP/GP ratio used in the data presented in Figure 3 that the binding is limited by the nanoparticle concentration. At lower cPS-NP/GP ratio (10 : 1 or 1 : 1), we measured binding efficiencies higher than 95%. It was not possible to measure binding at cPS-NP/GP ratios higher than 100 : 1 due to inefficient separation of the excess unbound cPS-NPs from the GP pellets. 

**Figure 4:**
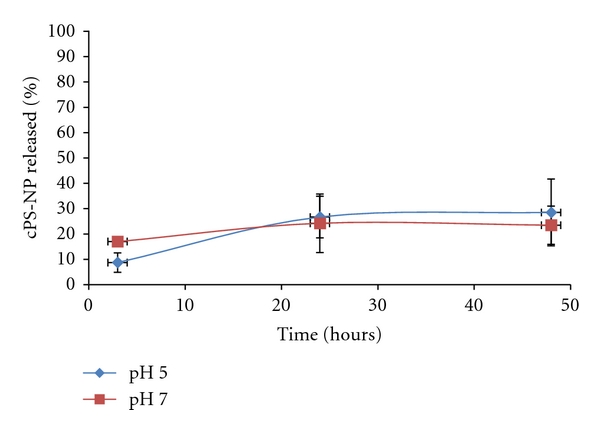
Stability of cPS-NP-GP. cPS-NP released from PEI-GP samples following incubation in PBS + 10% FBS (pH 7) or 0.1 M acetate buffer +10% FBS (pH 5).

cPS-NP-PEI-GP samples were evaluated for nanoparticle-binding stability at pH 5 and pH 7 in buffers containing 10% fetal bovine serum (FBS) to simulate cell uptake conditions. Fluorescence measurements of the released nanoparticles into solution and microscopic evaluation of the samples after 48 h incubation showed that more than 60% of the anionic nanoparticles remained bound to the modified PEI-GP particles (Figure 4). The stability of the electrostatic binding of cPS-NP to PEI-GPs provides for efficient glucan-mediated uptake of the cPS-NP-PEI-GPs into cells expressing glucan receptors (Figure 6). 

**Figure 5:**
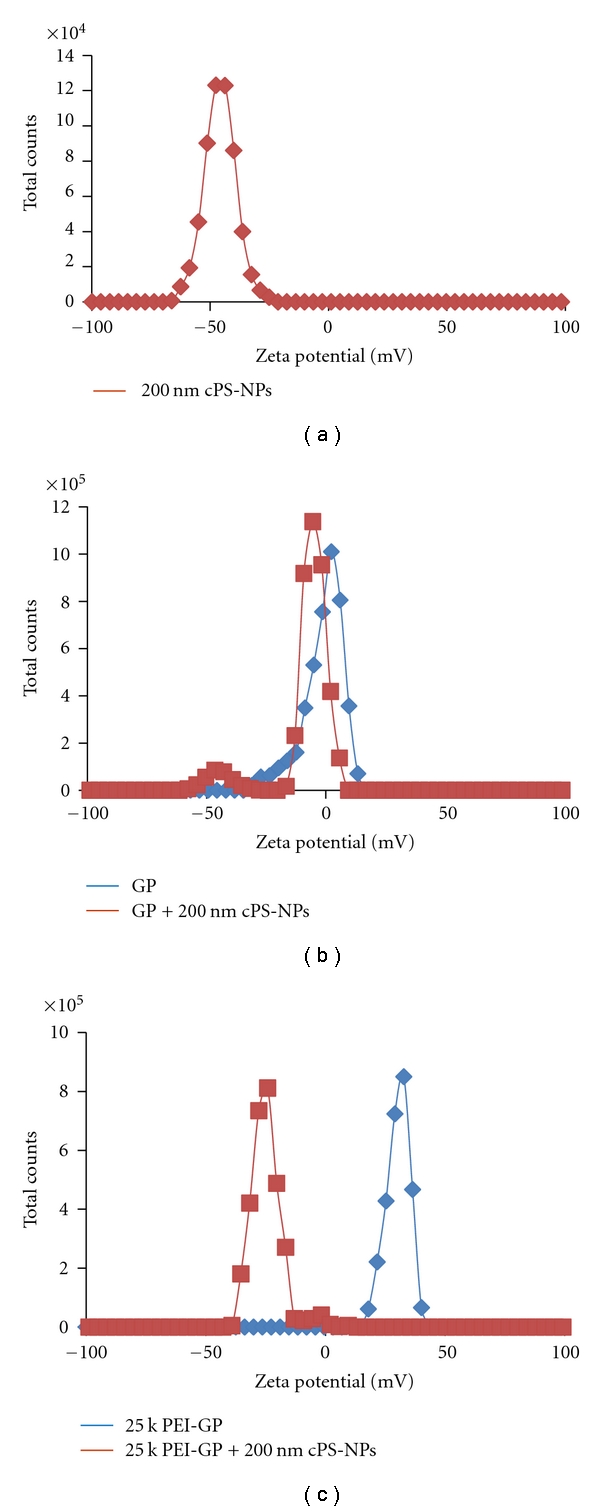
Zeta potential of (a) 200 nm anionic polystyrene NPs alone, (b) glucan particle (GP) control + cPS-NPs, and (c) 25 k PEI-GP + cPS-NPs.

Zeta potential was also used to demonstrate the binding of the anionic cPS-NPs to cationic PEI-GPs. The zeta potential data in Figure 5 shows the binding of cPS-NPs to cationic GPs as the zeta potential of the cationic GPs shift to an anionic value (−25 mV). Further, the effective separation of unbound cPS-NPs from the cPS-NP-GP sample is clear as there is only one peak (cPS-NP-GP) and no evidence of unbound cPS-NPs at ~−50 mV. In contrast, the zeta potential of the unmodified control GPs did not significantly shift following incubation with 200 nm cPS-NPs. 

**Figure 6:**
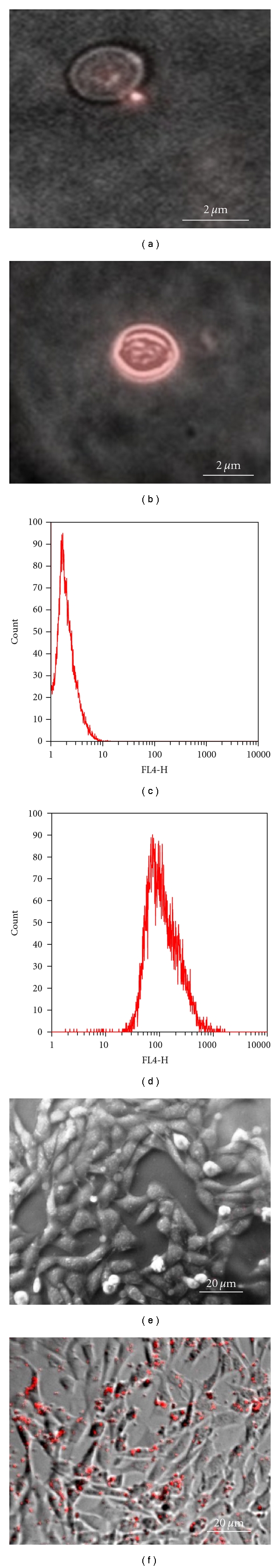
Microscopic images of (a) GP + 200 nm cPS-NPs and (b) 25 k PEI-GP + 200 nm cPS-NPs. FACS results of (c) GP + 200 nm cPS-NPs and (d) 25 k PEI-GP + 200 nm cPS-NPs. Microscopic images showing NIH 3T3-D1 uptake of (e) GP + 200 nm cPS-NPs and (f) 25 k PEI-GP + 200 nm cPS-NPs.

Microscopic evaluation (Figures 6(a) and 6(b)) of these samples confirmed the binding of the fluorescent nanoparticles to the surface of PEI-GPs with the cPS-NP fluorescence localized around the perimeter of the GP shells. Unmodified control GPs did not show fluorescent cPS-NPs rosetting the GPs. Flow cytometry was used to quantitate the binding of fluorescent cPS-NPs to unmodified GP and PEI-GPs. The results, shown in Figures 6(c) and 6(d), confirmed the fluorescent cPS-NP binding to the PEI-modified GPs. Although the number of 200 nm nanoparticles that can be bound to the surface of modified GPs (~70 cPS-NP/GP) is significantly less than the number of 20 nm particles that can be trapped inside GPs (>30,000 20 nm cPS-NP/GP), these two NP formulation strategies allow the use of GPs for the targeted drug delivery of drug-nanoparticle conjugates over a wide range of NP sizes and surface chemistries. Figures 6(e) and 6(f) show NIH3T3-D1 uptake of cPS-NP nanoparticles bound to 25 k PEI-GP. These particle uptake experiments demonstrate that cPS-NP electrostatically bound to PEI-GPs are more efficiently delivered to phagocytic cells than cPS-NP nanoparticles incubated with the control GP particles or free cPS-NPs. Additionally, the cell uptake experiments using cPS-NP-PEI-GP samples confirmed the stability results (Figure 3) of the electrostatically bound samples and that the low level of PEI surface modification of GPs, or the binding of anionic nanoparticles to the PEI-GP surface had no apparent impact on glucan-mediated phagocytosis or cellular toxicity.

### 3.2. Use of Glucan Particles for the Delivery of Mesoporous Silica Nanoparticles Loaded with Doxorubicin

Many types of nanoparticles have been used for drug delivery and imaging (i.e., silica nanoparticles, carbon nanotubes, gold nanoparticles, polymeric nanogels, magnetic iron oxide nanoparticles, quantum dots, and PLGA nanoparticles [1, 2, 39]). The drug can be physically trapped within nanoparticles, or chemically bound to the surface of the NPs. Methods have also been developed to precisely control particle size. We chose to study mesoporous silica nanoparticles (MSN) as a model system with GPs because of their ease of synthesis and ability to trap chemotherapeutic drugs (i.e., doxorubicin). A MSN sample containing tetraethoxyorthosilicate (TEOS), amino-propyltriethoxysilane (APTS), and 3-trihydroxysilylpropyl methylphosphonate was synthesized following the procedure reported by Lu et al. [32, 33]. The MSN was synthesized by a co-condensation method and the phosphate compound selected to have a larger alkyl chain than APTS to provide a particle with the outermost surface groups corresponding to anionic phosphate. This prevents aggregation of MSNs from interparticle hydrogen bonding between surface silanol groups and amine groups. Successful synthesis of this MSN sample was confirmed by zeta potential (−31.1 ± 5 mV) and DLS particle size measurements (MSN average size of 120 nm, polydispersity index PDI of 0.4). The broad particle size distribution and large particle size prevented the use of MSNs for loading inside GPs. However, the sample contained anionic phosphate groups allowed for electrostatic binding to the surface of cationic PEI-GPs. 

MSN was loaded with the chemotherapeutic doxorubicin (Dox), an anthracycline-type antitumor drug that exerts its antiproliferative activity via DNA intercalation and inhibition of DNA synthesis leading to cell death [40, 41]. Limitations in the use of Dox as an antitumor agent include chronic or acute cardiotoxicity. Dox has been studied using different nanoparticle delivery systems to enhance Dox delivery, minimize dosage, and reduce toxicity leading to the successful development of a liposomal Dox formulation (Doxil). 

**Figure 7:**
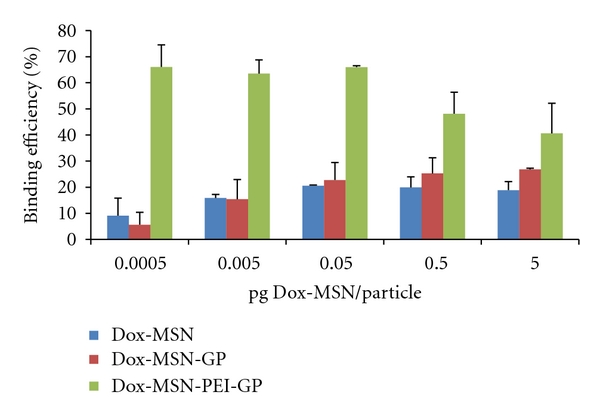
Binding efficiency of Dox-MSN binding to 25 k PEI-GP (the results are the average of three samples).

**Figure 8:**
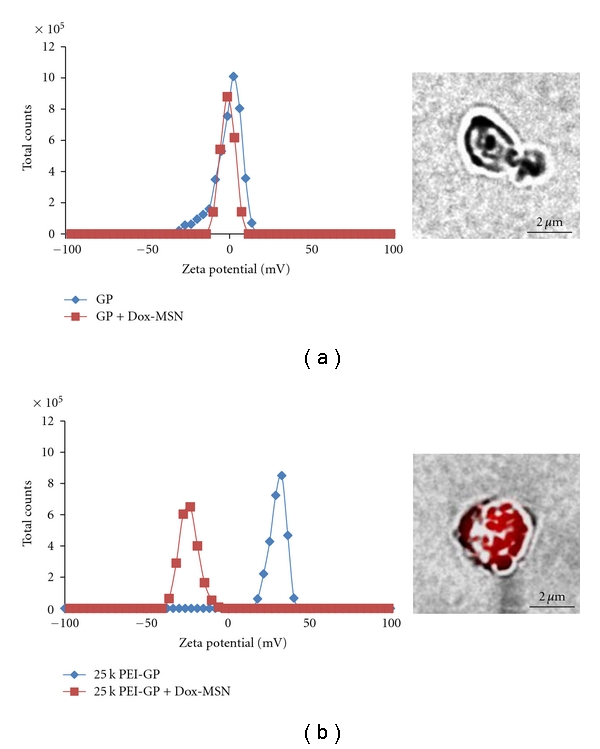
Zeta potential monitoring of Dox-MSN binding to (a) GP and (b) 25 k PEI-GP. Inset, fluorescent microscopic images of Dox-MSN bound to GP (a) and 25 k PEI-GP (b). Samples were prepared with 25 fg Dox-MSN/GP (~0.9 pg Dox/GP).

Dox was loaded into MSNs in DMSO at target concentrations ranging from 0 to 2 *μ*mol Dox/mg MSN. Following MSN Dox loading, the Dox-MSN samples were washed to remove unbound Dox. The amount of Dox loaded into the MSN samples was quantified by measuring Dox fluorescence extracted in methanol, and the results showed that the binding of Dox to MSN was 0.06 *μ*mol Dox/mg MSN, representing 3% of the input load. This binding is ~5 times higher than Dox binding to a control MSN sample without phosphate groups. Other groups have reported similar Dox-binding capacities with phosphonate functionalized MSNs (6–8% w/w) and have shown higher binding to these functionalized MSNs compared to MSN controls [42]. The Dox-MSN samples were stable in PBS (pH 7) enabling the electrostatic binding of anionic Dox-MSN to the surface of cationic PEI-GPs. This binding reaction was monitored by a fluorescence-binding assay (Figure 7), zeta potential (Figure 8), and confirmed by fluorescent microscopy (Figure 8, inset). The fluorescence-binding assay showed efficient and selective binding of Dox-MSN to PEI-GP at low Dox-MSN concentrations. Higher concentrations showed a reduction in binding efficiency likely due to saturation of available PEI for binding of Dox-MSN. The level of background binding of Dox-MSN nanoparticles to unmodified GPs corresponds to the fraction of nanoparticles that were not efficiently separated from the glucan particles. This is seen in Figure 7, both Dox-MSN alone and Dox-MSN with GP showed similar binding as a result of measuring the fluorescence of Dox-MSN-free nanoparticles in the pellet fraction. Dox-MSN-PEI-GP samples were incubated in 0.9% saline, PBS with 10% fetal bovine serum (FBS), and sodium acetate buffer (pH 5) at 37°C for 24 h and evaluated by fluorescence microscopy to confirm stability of Dox-MSN binding to PEI-GP. 

Zeta potential measurements confirmed the selective binding of Dox-MSN to PEI-GP. Dox-MSN had a negative zeta potential corresponding to the outer phosphate groups of MSN. Binding of anionic Dox-MSN to cationic PEI-GP shifted the zeta potential of the PEI-GP sample from a positive to a negative value. In comparison, the zeta potential shift of the GP sample is minimal confirming that Dox-MSN does not bind to unmodified GPs. Fluorescent microscopy confirmed that Dox-MSN binds to cationic PEI-GP but not GPs. 

**Figure 9:**
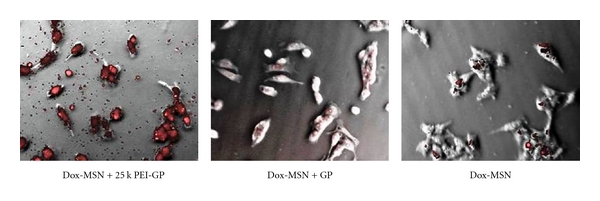
Efficient GP-mediated Dox delivery into NIH 3T3-D1 (pictures were taken at 40x magnification).

The Dox-MSN-GP and Dox-MSN-PEI-GP samples were tested for intracellular Dox delivery, and antiproliferative and cytotoxic activities in the NIH 3T3-D1 cell line. This cell line has been genetically modified to express the Dectin-1 glucan receptor and efficiently phagocytoses GPs.  Figure 9 shows that Dox-MSN-PEI-GP more effectively delivered Dox into NIH3T3-D1 cells than Dox-MSN-GP or Dox-MSN after 3-hour incubation. 

**Figure 10:**
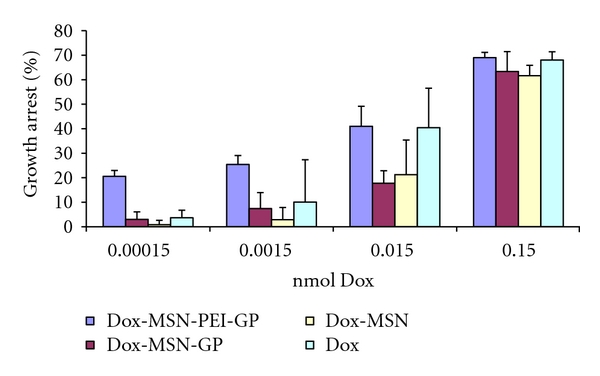
Growth arrest of NIH 3T3-D1 cells with Dox formulations (experimental results are the average of four samples).

Cells were incubated with varying concentrations of Dox-MSN-PEI-GP, Dox-MSN-GP, free Dox-MSN, or free Dox to assess antiproliferative and cytotoxic activities over 48 hours of incubation. Unincorporated materials were washed away after 3 hours of incubation, a sufficient period of time for efficient GP uptake by NIH 3T3-D1 cells, and the growth and viability of the cells were followed. High concentrations of free Dox or Dox-MSN (>2.5 *μ*g Dox-MSN containing ~0.15 nmol Dox) inhibited cell growth (>60%). As seen in Figure 10 at a concentration of 0.015 nmol Dox the Dox-MSN-PEI-GP samples showed similar effect as free Dox, but free Dox-MSN or Dox-MSN-GP samples showed less growth inhibition. Below the minimum inhibitory concentration (MIC) of free Dox (0.0015 nmol Dox), there is still a significant growth inhibition (20–30%) by the Dox-MSN-PEI-GP formulation (Figure 10) showing the increased efficacy of GP-targeted delivery of Dox-MSN-PEI-GP. Other groups have reported the use of MSN for delivery of other chemotherapeutics (i.e., camptothecin) and showed a 10-fold reduction in the drug concentration compared to free drug to achieve 50% cell death [32, 33]. The use of PEI-GP for Dox-MSN delivery allows for a reduction in drug dosage compared to free Dox or Dox-MSN demonstrating the advantage of GP-targeted delivery. Future work will focus on optimization of Dox incorporation into nanoparticles and controlled release of Dox from MSN and other nanoparticles (i.e., gold nanoparticles) to improve cytotoxicity of Dox NP-GP formulations. These formulations will be evaluated in dividing tumor cells (i.e., NIH 3T3-D1) and nondividing primary macrophages, and in cocultivation macrophage-tumor cell assays to test the hypothesis that macrophages can act as cell-based carriers of GP-NP-Dox formulations into tumors. Optimal GP-Dox NP-GP formulations will be tested *in vivo* for targeted macrophage Dox delivery and accumulation in tumors.

## 4. Conclusions

We have developed two strategies for the targeted delivery of nanoparticles into phagocytic innate immune cells. Nanoparticles of less than 30 nm in diameter were encapsulated within the hollow cavity of GPs (~36,000 NPs/GP for 20 nm NPs). Larger anionic nanoparticles (>100 nm) were electrostatically bound to the surface of GPs derivatized with the cationic polymer PEI allowing for delivery of ~70 NPs/GP. Mesoporous silica nanoparticles (120 nm) containing doxorubicin were electrostatically bound to PEI-GPs providing for the targeted delivery of the Dox-MSN-PEI-GPs to cells capable of phagocytosing glucan particles. At Dox levels below an effective free-drug concentration, an equivalent amount of Dox-MSN-PEI-GPs efficiently delivered sufficient Dox to inhibit the growth of the GP-phagocytic cell line NIH 3T3-D1. These results demonstrate that the NP-GP delivery system offers the potential of GP-mediated macrophage-targeted delivery of multiple nanoparticles in a single uptake event providing for high efficiency intracellular drug delivery. The possibility that macrophages can serve as Trojan horses carrying and releasing the drug into solid tumors may further enhance the *in vivo* Dox-MSN-PEI-GP antitumor effect. We are currently studying a variety of drug loaded nanoparticulate formulations that may provide advantages of higher drug binding capacity and the possibility of controlled release. In addition, the use of certain types of nanoparticles (i.e., gold nanoparticles, magnetic iron oxide) may add theranostic properties to the NP-GP delivery system.

## Figures and Tables

**Table 1:** PEI surface functionalization results and zeta potential values of GP and PEI-GPs.

GP sample	PEI surface functionalization results *μ*mol PEI/mg GP	Zeta potential peak (±5 mV)
GP	—	2.4
1.2 k PEI-GP	0.012 ± 0.002	22.1
1.8 k PEI-GP	0.031 ± 0.021	21.7
10 k PEI-GP	0.015 ± 0.001	21.1
25 k PEI-GP	0.0136 ± 0.003	30.2
100 k PEI-GP	0.0192 ± 0.001	33.3
